# Recurrent Early Thrombus Formation in HeartMate II Left Ventricular Assist Device

**DOI:** 10.1177/2324709613490676

**Published:** 2013-05-20

**Authors:** Massimo Capoccia, Christopher T. Bowles, Anton Sabashnikov, Andre Simon

**Affiliations:** 1 Royal Brompton & Harefield NHS Foundation Trust Harefield Hospital, Middlesex, UK

**Keywords:** mechanical circulatory support, left ventricular assist device (LVAD), heart transplantation, thrombus

## Abstract

Left ventricular assist devices are becoming an established treatment for end-stage heart failure. In spite of their proven benefit, pump thrombosis remains a significant complication. Here we describe the challenging management of a patient with recurrent pump thrombosis.

## Introduction

In spite of continuing improvements in ventricular assist device (VAD) technology, anticoagulation management remains a significant challenge. Of the devices currently available, the HeartMate II (HMII; Thoratec Corporation, Pleasanton, CA) has been the most widely used second generation axial flow pump with more than 10 000 devices implanted so far.^1^ Although there is evidence that the HMII is associated with superior clinical efficacy with respect to earlier designs and is considered to be the gold standard by some, early thrombus formation has been described.^2^ Here we report a case of recurrent thrombus formation 10 months after HMII implantation and subsequently 2 months after device replacement.

## History

A 30-year-old gentleman with a diagnosis of dilated cardiomyopathy and severe left ventricle (LV) systolic dysfunction was referred to our unit for cardiac transplant assessment and was subsequently listed. Because of precipitous hemodynamic deterioration, the decision was made to use an LVAD as a bridge to transplantation, but implantation had to be delayed for 6 weeks because of pulmonary embolism. During this period, he deteriorated and required veno-arterial extracorporeal membrane oxygenation. Three days later, he underwent HeartMate II LVAD implantation with simultaneous Levitronix right ventricular assist device (RVAD; Levitronix LLC, Waltham, MA) support. The RVAD was explanted after 13 days, and he was discharged home 38 days later. He remained reasonably well on follow-up until he required hospital readmission 10 months later due to syncope, extreme shortness of breath, and hypotension. Elevated plasma-free hemoglobin and an abnormal device sound were suggestive of pump thrombosis.^3^ Initial management consisted of aggressive anticoagulation and thrombolytic treatment with Tirofiban, which was complicated by the onset of a severe left arm hematoma causing compartment syndrome requiring fasciotomy and neurolysis. Right heart catheterization demonstrated acceptable hemodynamic measurements. A multidisciplinary meeting decision was made to replace the HMII with a device of similar design using the original inflow cannula and outflow graft, the patency of which had been previously confirmed by computed tomography. Although the procedure was surgically successful, the early postoperative course was complicated by bleeding and the development of coagulopathy requiring reexploration. In addition, significant inotropic support and nitric oxide were commenced but no RVAD support was needed this time. Finally, he made a slow but uneventful recovery. He was discharged home 21 days following his pump replacement.

Two months after discharge, he was admitted to his district hospital with signs of severe dehydration secondary to the onset of diarrhea and vomit 1 week earlier. Fluid resuscitation was commenced but eventually continuous veno-venous hemofiltration was required because of renal dysfunction. Seven days later, he was transferred to our intensive care unit for further management. Severe liver failure was observed with deranged coagulation and no evidence of liver disease making the clinical picture suggestive of low output syndrome. Although pump thrombus could not be diagnosed by computed tomography, echocardiographic features suggested otherwise: possible thrombus on the orifice of the inflow cannula and aortic valve opening every beat. An LVAD ramp test was performed.^4^ Increasing the pump speed from the baseline of 9200 to 11 000 rpm neither resulted in the predicted increase in pump flow/power nor the loss of arterial pulsatility suggestive of impaired flow increase and ineffective left ventricular decompression as a result of LVAD speed increase. Interestingly, the LVAD power and estimated flow were within normal limits (7.1 W, 6 L/min, respectively). Despite aggressive treatment, no improvement was achieved and he was deemed unsuitable for further surgery. Palliative care was implemented and he died ten days after admission. The explanted device showed evidence of impeller thrombosis and blood path occlusion.

**Figure 1.**
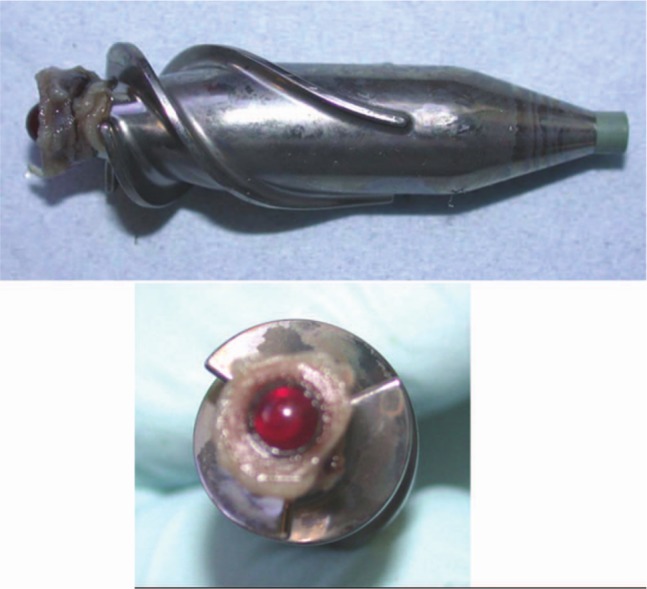
Frontal and lateral views of the impeller: visible thrombus around the ruby bearing.

## Discussion

Rotary LVAD pump thrombosis remains a significant ubiquitous complication and has an incidence in the range 0.01 to 0.1 events per year.^5^ It is likely that events are underreported because of spontaneous resolution, successful treatment, or successful bridging to transplantation in spite of ongoing pump thrombosis. Data from our institution suggest that the risk of pump thrombosis is relatively high in the early postoperative period and this may be attributable to the risk of particulate ingress (thrombus, surgical debris) at the time of surgery, stabilization of both anticoagulation therapy and the interface between the blood and the device.

**Figure 2.**
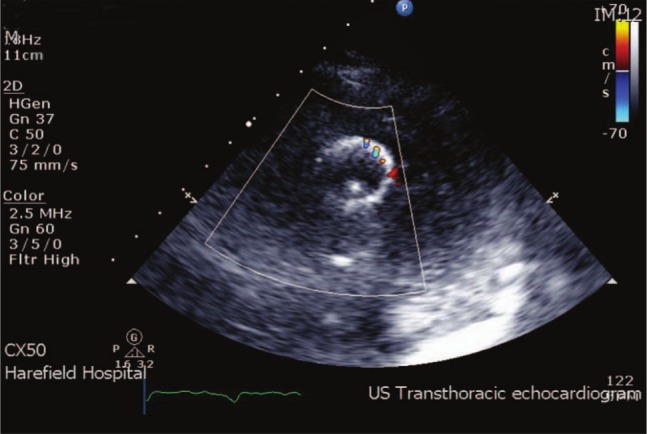
Echocardiographic assessment during second admission: image suspicious for pump thrombus.

The definitive diagnosis of LVAD thrombosis remains a significant challenge. Diagnostic imaging methods are unable to reveal thrombus deposition within the LVAD. Echocardiography can offer indirect evidence under some circumstances, including LV thrombus resulting in inflow cannula occlusion and insensitivity of LV dimensions to pump speed change. Alterations in biochemical markers such as plasma-free hemoglobin and lactate dehydrogenase had clinical utility in this case and in previous reports.^2^ This case shows how normal pump power and estimated flow can provide a false sense of security under conditions of suboptimal perfusion presumably as a result of increased drag on the impeller resulting in an overestimation of pump flow. LVAD acoustic waveform analysis has been developed in our center^3^ with the aim of assisting in the diagnosis and management of pump thrombosis. This approach entails recording the LVAD sound and the electrocardiogram simultaneously and analyzing the waveforms with proprietary software scripts (Matlab). This method shows that the acoustic profile is primarily determined by pump speed, impeller symmetry, and the interaction between the native LV and the LVAD. Aberrant acoustic profiles have been detected in Heartware HVAD pump thrombosis, which normalize following successful anticoagulation therapy. Another useful investigation under conditions of suspected pump thrombosis is the ramp test that was applied in this case.^4^

## Conclusion

Successful treatment of pump thrombosis is likely to depend on prompt diagnosis and on implementation of an appropriate treatment strategy, which is likely to be device specific. This case highlights the difficulty of managing pump thrombosis in spite of aggressive therapeutic measures.
